# Commentary: Neuronal regulation of type 2 innate lymphoid cells via neuromedin U

**DOI:** 10.3389/fphar.2018.00230

**Published:** 2018-03-14

**Authors:** Geraldine M. Jowett, Joana F. Neves

**Affiliations:** ^1^Department of Craniofacial and Regenerative Biology, King's College London, London, United Kingdom; ^2^Department of Experimental Immunobiology, School of Immunology and Microbial Sciences, King's College London, London, United Kingdom

**Keywords:** innate lymphoid cells, neuromedin U, IBD, mucosal epithelia, neuroimmunology

The links between the intestine and the nervous system have gained renewed attention since diverse factors such as diet, the microbiota, inflammation, and drugs are now known to not only influence the function of this gut-brain axis, but also have systemic effects. Recent studies add a new contributor to this mix, revealing that mucosal neurons can sense helminth infections and directly activate group 2 innate lymphoid cells (ILCs) opening the door to new therapeutic strategies to tackle intestinal dysregulation (Figure 1).

**Figure 1:**
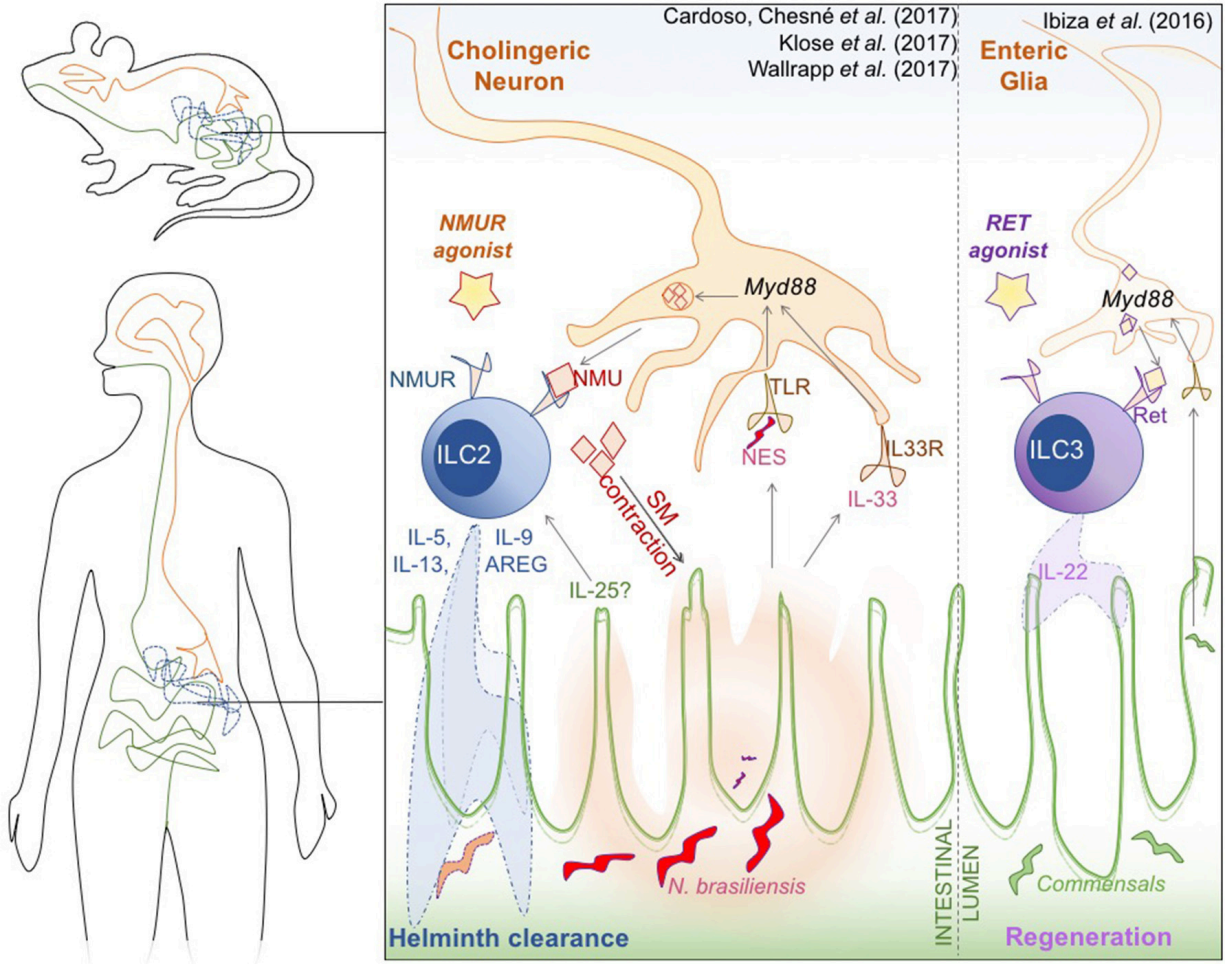
Novel neuro-immune interactions in the mammalian gut. Cholinergic neural cells, type 2 innate lymphoid cells (ILC), and the intestinal epithelium orchestrate an innate response to *N*. *brasiliensis* worm infection **(Left)**. Glial cells sense alarmins in the form of the helminth *N. brasiliensis* excretory/secretory products (NES) and the cytokine IL-33, that is secreted by damaged epithelium. This process is mediated by Myd88, and triggers the production and secretion of Neuromedin U (NMU). NMU induces smooth muscle contractions around the epithelium, which is hypothesized to help physically expulse the helminth infection, but is also recognized by the *NMURI receptor* specifically expressed on ILC2. NMU induces *Ki67* mediated proliferation and activation of ILC2, which secrete type-2 cytokines [IL-5, IL-9, Il-13, amphiregulin (AREG)] to promote clearance of helminthic infection. A similar Myd88-dependent neuronal activation of ILC3 occurs when commensal microbiota induce neuronal activation of *Ref*-expressing ILC3 **(Right)**, triggering the characteristic release of cytokine IL-22 to promote epithelial regeneration. Both receptors could be attractive therapeutic targets to modulate intestinal inflammation in IBD.

Innate lymphoid cells (ILCs) are highly abundant in mucosal tissues and have attracted interest due to their role in controlling bacterial and parasitic infections, in amplifying immune signals, and in maintaining intestinal barrier integrity. For example, group 2 ILC (ILC2) have a key role in the immune response to helminth infection and group 3 ILC (ILC3) are important for intestinal epithelial regeneration (Britanova and Diefenbach, 2017). Furthermore, this seems to be a bidirectional relationship, as epithelial Tuft-cell derived IL-25 appears to be crucial for ILC homeostasis, particularly of ILC2 (von Moltke et al., 2015; Gerbe et al., 2016; Howitt et al., 2016). Despite these protective roles, ILC can also promote intestinal inflammation, as evidenced by the accumulation of group 1 ILC (ILC1) in the gut of patients with inflammatory bowel disease (IBD) (Bernink et al., 2013). The intestinal epithelium and ILC thus clearly exist in a delicate balance, and together with the luminal commensal microbiota form a trinity that is essential maintaining intestinal homeostasis. Recent publications in Nature reveal that neural cells are also crucial for this homeostatic balance, not only through interactions with the epithelium and the microbiota, but by directly engaging ILC2.

Cardoso et al. (2017) demonstrate that both murine and human ILC2 express a receptor that recognizes the neuropeptide neuromedin U (NMU). NMU is a highly conserved 33 amino-acid peptide secreted by cholinergic neurons that mediate intestinal peristalsis and modulate the sense of satiety (Minamino et al., 1985; Martinez and O'Driscoll, 2015). The authors have shown that within immune cells, the expression of NMU-receptor NMUR1 is enriched in ILC2, and that NMUR1-expressing ILC2 closely associate with NMU-expressing neurons both in the lung and in the gut. Both *in vitro* and *in vivo* stimulation of ILC2 with recombinant NMU led to the release of amphiregulin (AREG) and IL-5 and IL-13 cytokines which are associated with immune defense against helminths (Fallon et al., 2006). Thus, to probe the physiological role of NMU-driven activation of ILC2, the authors challenged mice with the helminth *Nippostrongylus brasiliensis*. Notably, infection resulted in increased expression of NMU in wild-type mice, and both NMUR1 KO mice or chimeric mice in which ILC2 do not express NMUR1 were significantly less able to clear *N. brasiliensis*.

Finally, the group also shed light on the mechanism underlying activation of this neuron-immune axis during infection. Using enteric neuronal organoids, they found that neuronal expression of *Nmu* in neurons was triggered by both IL-33 and *N. brasiliensis* excretory-secretory products (NES), and was dependent on Myd88. On the ILC2, NMUR1 activation triggered ERK1/2 phosphorylation and a Ca^2+^-calcineurin–NFAT cascade that results in cytokine production. The authors propose that Myd88 is a signaling adaptor of both TLR (which could sense NES) and the IL-33R (ST2) expressed by neurons, allowing these cells to directly sense infection, thereby triggering NMU secretion and ILC2 activation. The same group had previously demonstrated that enteric glial cells can also sense commensal products in a *Myd88* dependent manner and that ILC3 express the neuro-receptor *Ret*, secreting IL-22 in response to glial signaling, thereby mediating epithelial repair, a mechanism similar to that of NMU-NMUR(ILC2) pathway (Ibiza et al., 2016).

Klose et al. independently recapitulated many aspects of the neuron-ILC2 axis, including NMUR1 expression on ILC2, co-localization between ILC2 and neurons, secretion of IL-5, IL-9, and IL-13 by ILC2 upon NMU activation and increased worm burden in NMUR1 KO mice. These effects were eosinophil independent as shown by NMU activation of ILC2 in eosinophil-deficient animals (Klose et al., 2017).

In a third paper, the role of IL-25 for NMU function in ILC2 was addressed by showing that together these signals augment allergic inflammation in the lung in response to house dust mites (Wallrapp et al., 2017). Since IL-25 is secreted by intestinal Tuft cells and is important for ILC2 homeostasis in the intestine, it will be interesting to investigate whether this synergistic effect also occurs in the gut. Future studies should also address if NMU's roles in ILC2 activation and smooth muscle contraction are synergistic, allowing mucosal tissues to physically expel pathogens, as suggested by Cardoso et al. (2017).

Collectively, these papers illustrate why enteric neuronal cells and their involvement in regulating the intestinal milieu have become of interest to immunologists. However, it is important to keep in mind that the gut also signals back to the brain. For example, enterochromaffin enteroendocrine cells (EEC) in the intestinal epithelium are capable of sensing commensal bacterial products and trigger the secretion of serotonin into the synaptic gap between these EEC and neurons (Bellono et al., 2017). Therefore, it will be exciting to determine whether upon neuronal-mediated activation during infection, the gut signals back to the brain, and its' consequences. More importantly, it will be key to dissect if the gut-brain axis, the epithelial-ILC axis, and the neuro-immune axis can be tied together, elucidating whether these connections operate redundantly, in parallel, or perhaps sequentially.

This is a fast-moving field, and combining these axes to better understand ILC activation in mucosal tissues could open many pharmacological doors. For example, it is plausible that other ILC subsets, including pathogenic IFNγ-secreting ILC1, are similarly triggered by neuropeptides, which would provide a source of therapeutic targets for IBD (Bernink et al., 2013). NMUR and RET also provide interesting targets for agonists that could help locally alleviate inflammatory signals. With the constant improvements in organoid culture systems and transcriptomic techniques, systems could be established in the near future to screen for novel molecules that act on targets in human neural, intestinal, and immune cells, leading to new treatments for IBD.

## Author contributions

GJ: Wrote and revised the commentary and executed the figure; JN: Designed, wrote, and revised the commentary and the figure.

### Conflict of interest statement

The authors declare that the research was conducted in the absence of any commercial or financial relationships that could be construed as a potential conflict of interest.
